# Ginsenoside CK, rather than Rb1, possesses potential chemopreventive activities in human gastric cancer *via* regulating PI3K/AKT/NF-κB signal pathway

**DOI:** 10.3389/fphar.2022.977539

**Published:** 2022-09-29

**Authors:** Yan Wan, Dong Liu, Jia Xia, Jin-Feng Xu, Li Zhang, Yu Yang, Jiao-Jiao Wu, Hui Ao

**Affiliations:** ^1^ State Key Laboratory of Southwestern Chinese Medicine Resources, Chengdu University of Traditional Chinese Medicine, Chengdu, China; ^2^ Innovative Institute of Chinese Medicine and Pharmacy, Chengdu University of Traditional Chinese Medicine, Chengdu, China

**Keywords:** ginsenoside Rb1, ginsenoside CK, gastric cancer, network pharmacology, apoptosis, gut microbiota

## Abstract

Ginsenoside Rb1, a main component of ginseng, is often transformed into ginsenoside CK by intestinal flora to exert various pharmacological activity. However, it remains unclear whether ginsenoside CK is responsible for the anti-gastric cancer effect of ginsenoside Rb1 *in vivo*. In this study, network pharmacology was applied to predict the key signal pathways of ginsenoside Rb1 and ginsenoside CK when treating gastric cancer. The anti-proliferative effects of ginsenoside Rb1 and ginsenoside CK and the underlying mechanism in gastric cancer cells were explored by MTT, Hoechst3328 staining, ELISA, RT-qPCR and Western blotting. The results showed that PI3K-AKT/NF-κB signal pathway was the common important pathway of ginsenoside Rb1 and CK in the treatment of gastric cancer. The results of MTT assay showed that ginsenoside Rb1 could hardly inhibit the proliferation of HGC-27 cells, whereas ginsenoside CK could inhibit the proliferation of HGC-27 cells. Hoechst3328 staining showed that cells in the ginsenoside CK group were densely stained bright blue and nuclear fragmented, indicating that apoptosis occurred. ELISA results showed that ginsenoside CK could effectively downregulate the levels of cyclin CyclinB1 and CyclinD1, but ginsenoside Rb1 had no significant effect. Also, the results of Western blot and RT-qPCR showed that ginsenoside CK inhibited the expressions of anti-apoptosis-related protein Bcl-2 and apoptosis-related pathway PI3K/AKT/NF-κB, and promoted the expression of pro-apoptosis proteins Bax and Caspase 3, whereas ginsenoside Rb1 exerted no effect. In short, ginsenoside Rb1 had no anti-gastric cancer cell activity *in vitro*, but ginsenoside CK could effectively inhibit cell proliferation and induce cell apoptosis in HGC-27 cells. The mechanism might relate to the inhibitory effect of ginsenoside CK on the PI3K/AKT/NF-κB pathway. These results suggest that ginsenoside CK might be the *in vivo* material basis for the anti-gastric cancer activity of ginsenosides.

## 1 Introduction

Gastric cancer is the fourth most common male cancer diagnosis in the world, after lung, prostate and colorectal cancer, and the fifth among women, after breast cancer, colorectal cancer, cervical cancer, and lung cancer. The pathogenesis of gastric cancer involves multi-steps, multi-factors and multi-targets. It is estimated that the fatality rate of gastric cancer is about 70%, which is much higher than other epidemic diseases (Sung et al., 2021). At present, the main treatment of gastric cancer is the combination of neoadjuvant radiotherapy and chemotherapy, molecular targeted therapy and immunotherapy (Song et al., 2017). However, these methods have defects that cannot be ignored. For example, although radiotherapy and chemotherapy are effective treatments, serious side effects (loss of appetite, indigestion, burning sensation, nausea, vomiting, etc.) seriously affect the efficiency of treatment (Bae et al., 2017). Natural products, which are extracted from the plant kingdom with the characteristics of low toxicity and few side effects, are multiple targeted (Wan et al., 2021). Therefore, it is of great importance to discover natural drugs with anti-gastric cancer effects.

Ginseng is known as the “King of Herbs,” which has the effect of nourishing vitality, strengthening the body and eliminating evil, according to Traditional Chinese Medicine. As the main active ingredients of ginseng, ginsenosides play a pivotal role in the pharmacological effects of ginseng. Current studies have shown that total ginsenosides have a certain anti-gastric cancer activity, whereas ginsenoside Rb1, one of the main prototype components of total ginsenosides, also plays an important role in anti-gastric precancerous lesions, indicating that ginsenoside Rb1 may have anti-gastric cancer potential (Xu et al., 2018a). Notably, ginsenoside compound K (CK) is the gut microbiota-derived product of ginsenoside Rb1. Recently, a growing number of studies demonstrated that CK, the microbial transformed metabolites of Rb1, showed greater therapeutic activities compared with its parent compound Rb1, either *in vivo* or *in vitro*. For example, *in vitro* models of breast and colon cancer, ginsenoside CK has better anticancer activity than ginsenoside Rb1 (Wang et al., 2012; Yao et al., 2018). Therefore, it is worth studying whether ginsenoside CK has stronger anti-gastric cancer activity than ginsenoside Rb1.

Also, the anti-cancer mechanism of ginsenoside Rb1 and CK is worth studying. However, the pathogenesis of gastric cancer is complex, involving multiple pathways and targets, which brings difficulties to the discovery of the anti-cancer drugs. As a systematic and comprehensive discipline, network pharmacology can easily obtain relevant targets for drug treatment of diseases, and select appropriate signaling targets for mechanism analysis. However, the network pharmacology results are only predictions and often need to be verified by the experiments. Therefore, it is undoubtedly a promising approach to study the anti-gastric cancer mechanism of ginsenoside Rb1 and CK based on the results of network pharmacology.

This study is designed to resolve the above problems according to the corresponding experimental scheme. The network pharmacology was applied to speculate the common signaling pathway responsible for the anti-gastric cancer effects of ginsenoside Rb1 and CK, and its pharmacological effect and the underlying mechanism were verified by the *in vitro* experiments. The purpose of this study is to compare the anti-gastric cancer activity of ginsenoside Rb1 and CK, and in turn explore the material basis and mechanism the anti-gastric cancer effect of ginsenosides *in vivo*, providing scientific basis for the clinical application of ginsenosides.

## 2 Materials

Ginsenoside Rb1 and ginsenoside CK were purchased from Chengdu Mansite Biotechnology Co., Ltd., and the purity was higher than 98%. (Chengdu, China). Human gastric cancer cell line HGC-27 was purchased from Shanghai Fuheng Biotechnology Co., Ltd. (Shanghai, China). DMEM, penicillin and streptomycin were purchased from Shanghai Biyuntian Biology Co., Ltd. (Shanghai, China). Fetal bovine serum was purchased from Zhejiang Tianhang Biotechnology Co., Ltd. (Zhejiang, China). MTT is purchased from Biosharp Company (Guangzhou, China). HumanCyclin-B1ELISAKIT and HumanCyclin-D1ELISAKIT are purchased from Ruixin Biotechnology Co., Ltd. (Fujian, China). 5X All-In-One MasterMix was purchased from abm (Canada). Bcl-2 antibody (AF6139), Bax antibody (AF0120), Phospho-I κB alpha antibody (AF 2002), Phospho-PI3K P85 alpha antibody (AF3241), Phospho-pan-AKT1/2/3 antibody (AF0016) and pan-AKT1/2/3 antibody (AF6261) were purchased from Affinity (Jiangsu, China). IκB alpha antibody (#9242), NF- κB p65 antibody (#8242) and Caspase-3 antibody (#14220) were purchased from Cell Signaling Technology (United States). PI3K p85alpha antibodies (TA6241) were purchased from Abimat Biomedical Co., Ltd. (Shanghai, China). Goat anti-rabbit IgG-HRP (Cat. No. 05-4030- 05) were purchased from Multi Sciences (LIANKE) Biotech Co., Ltd. (Hangzhou, China). Animal Total RNA Isolation Kit (R210801), RT EasyTM II (210401) and Real Time PCR EasyTM-SYBR Green I (P210501) were purchased from Chengdu Fuji Biotechnology Co., Ltd. (Chengdu, China). Hoechst33258 (C0020) was purchased from Beijing Solebo Technology Co., Ltd. (Beijing, China).

## 3 Methods

### 3.1 Study on the mechanism of ginsenoside Rb1 and its intestinal bacterial transformant-ginsenoside CK in the treatment of gastric cancer based on network pharmacology

#### 3.1.1 Target prediction of ginsenoside Rb1 and CK

The Herb Ingredients’ Targets (HIT) database is made up of more than 3,250 articles manually (Liang et al., 2019). In addition to more than 1,300 kinds of Chinese herbal medicine and 586 kinds of traditional Chinese medicine compounds, there are 1,301 protein targets. Compared with the traditional database, the information obtained by HIT database is calibrated by manual standardization. Therefore, the potential targets of ginsenoside Rb1 and CK were obtained by HIT (http://lifecenter.biosino.org/) database.

#### 3.1.2 Target prediction of gastric cancer

Using “gastric cancer” as the key word, the target of gastric cancer was obtained in disgenet (http://www.disgenet.org/), malacards (http://www.malacards.org/) and OMIM (https://omim.org/) database, and the obtained target was de-repeated to get the potential target of the disease.

#### 3.1.3 Core target “fishing” and protein-protein interaction network construction

BisoGenet aims to evaluate the prominence of functional relationships between genes or proteomes from proteomics or genomics experiments. A more comprehensive set of PPI networks can be obtained by expanding and analyzing the input targets through the internal integration database of Bisogenet. In the Cytoscape 3.8.2 software (Wang and Yuan, 2022), input the potential targets of ginsenoside CK, Rb1 and gastric cancer into the Bisogenet plug-in, click “Geneidentifiersonly” to enter the next step “DataSettings,” check the “ProteinProteinInteraction” option, and click OK to construct the PPI network of ginsenoside F2, Rd and gastric cancer, respectively. Then the PPI networks of ginsenoside Rb1 and CK were intersected with the PPI network of gastric cancer by Merged tool to obtain the PPI network of ginsenoside Rb1 for gastric cancer and the PPI network of ginsenoside CK for gastric cancer. Use the CytoNCA plug-in to calculate the attribute values of two groups of PPI networks. In the PPI network of ginsenoside Rb1 in the treatment of gastric cancer, the double median of Degree value was used to screen once, and then Degree, Betweenness and Clossness were used to screen twice to obtain the core target of ginsenoside Rb1 in the treatment of gastric cancer. In the PPI network of ginsenoside CK in the treatment of gastric cancer, the core target of ginsenoside CK in the treatment of gastric cancer was obtained by twice screening the median of Degree, Betweenness and Clossness.

#### 3.1.4 Gene ontology and pathway enrichment analysis

The core targets of ginsenoside Rb1 in the treatment of gastric cancer and ginsenoside CK in the treatment of gastric cancer were imported into the Metascape (http://metascape.org/) database, the species selection “*Homo sapiens*,” and click Costom Analysis to proceed to the next step. On the Enrichment page, select GO Biological Processes option for gene ontology (Gene Ontology, GO) biological process analysis, and check KEGG Pathway option for KEGG analysis. The results of GO analysis and KEGG enrichment were obtained.

### 3.2 Demonstration of the anti-gastric cancer mechanism of ginsenoside Rb1 and its intestinal bacterial transformant-ginsenoside CK regulating PI3K/AKT/NF-κB apoptosis pathway based on *in vitro* experiments.

#### 3.2.1 Cell culture

HGC-27 cells were cultured in DMEM enriched with 10% fetal bovine serum and 1% penicillin/streptomycin. The culture environment is an incubator under the conditions of 5% carbon dioxide and 37°.

#### 3.2.2 Cell viability assay

HGC-27 cell suspension (100 μl) in logarithmic growth phase was inoculated in 96-well plate at the rate of 5 × 10^−5^ per well. The cells were cultured for 24 h and then added with drug-containing medium. Experimental groups: Rb1 or CK (10, 20, 30, 40, 50, and 60 μM) acted on HGC-27 cells, the control group (with cells) added the same amount of drug carrier solvent (DMSO content <0.3%), and the blank group (no cells) only added the same amount of medium. Five multiple holes were set up for each dose, and cultured for 24, 48, and 72 h after adding the drug, then the medium was absorbed, and each well was added with MTT (5 mg/ml) 20 and 100 μl basic medium, and continued to culture for 4 h. The supernatant was absorbed, and 100 μl of DMSO was added to each hole to avoid light and oscillate 10 min, so that the crystal could be fully dissolved. The absorbance (OD) of each hole at 490 nm was measured by enzyme-linked immunosorbent assay (ElISA), the cell survival rate of each group (%) was calculated, the line chart was drawn, and the IC_50_ of the inhibitory effect of drugs on cells was calculated. The cell survival rate (%) was calculated according to the following formula: cell survival rate (%) = (OD value of administration group-OD value of blank group)/(OD value of control group-OD value of blank group) × 100%.

#### 3.2.3 Cell morphology observation

In order to observe the effect of drugs on cell morphology, HGC-27 cells were evenly inoculated in five petri dishes. After the cells grew to 70% Mel 80%, the cells were treated with different concentrations of ginsenoside CK (20, 40, and 60 μM) and ginsenoside Rb1 (60 μM) for 16 h, and the cell morphology of each group was observed under ×200 microscope.

#### 3.2.4 Apoptosis observation

HGC-27 is administered as described in Section 3.2.3. Then the cells were fixed with cell fixation solution, washed and removed properly after 30 min, and Hoechst33258 staining solution was added to cover the sample. Then the Hoechst33258 staining solution was removed and washed with PBS for 2-3 times. Finally, observed directly under the fluorescence microscope, if the cells are densely stained bright blue and show nuclear fragmentation, it indicates the occurrence of apoptosis.

#### 3.2.5 Enzyme-linked immunosorbent assay

HGC-27 is administered as described in Section 3.2.3. Then the supernatant of cell was collected and CyclinB1 and CyclinD1 were detected by enzyme linked immunosorbent assay (Elisa) kit.

#### 3.2.6 Real time quantitative PCR assay

Extract the total RNA by using Cell Total RNA Isolation Kit, according to the manufacturer’s instructions. The total RNA was reverse transcribed into cDNA with 5X All-in-One Master Mix. Quantitative PCR was carried out on the applied biological system 7900HT FAST system using SYBR Green PCR Master Mix. The RT-qPCR reaction conditions are as follows: 95°C for 10 min, 40 cycles of 95°C for 15 s, and 60°C for 30 s. The relative mRNA expression level was calculated by 2^−ΔΔCT^ method. The sequence of primers used by RT-qPC is shown in Table 1.

**TABLE 1:** Primer information.

Gene	Primer	Reverse
PI3K	Forward	GGT​TTG​GCC​TGC​TTT​TGG​AG
	Reverse	CCA​TTG​CCT​CGA​CTT​GCC​TA
AKT	Forward	GGA​CAA​GGA​CGG​GCA​CAT​TA
	Reverse	CGA​CCG​CAC​ATC​ATC​TCG​TA
NF-κB	Forward	AAT​GGG​CTA​CAC​CGA​AGC​AA
	Reverse	TTG​CGG​AAG​GAT​GTC​TCC​AC
NF-κB P65	Forward	TCC​TAT​AGA​AGA​GCA​GCG​TGG
	Reverse	GCC​AGA​GTT​TCG​GTT​CAC​TC
βactin	Forward	CCT​TCC​TGG​GCA​TGG​AGT​C
	Reverse	TGA​TCT​TCA​TTG​TGC​TGG​GTG

#### 3.2.7 Western blot assay

Treat the cells as described in Section 3.2.3. Then split it with RIPA cleavage solution on the ice. According to the manufacturer’s instructions, the BCA protein assay kit is used to quantify the protein concentration of each sample. After quantification, the protein sample buffer was added and heated at 100°C for 5 min. Then, each group of equal amount of protein was loaded into 10% SDS-PAGE and transferred to PVDF membrane. Then, the membrane and the primary antibodies (β-actin, PI3K, Akt, phospho-PI3K, phospho-AKT, p65, IκBα, p-IκBα, Bax, Bcl2, and caspase 3; 1:1,000) were used overnight at 4°C and the secondary antibodies (Goat anti-Rabbit IgG-HRP; 1:5,000) was used at 37°C for another 2 h. Finally, the protein bands were visualized by ECL Kit, and the immunoblotting signals were quantitatively analyzed by ImageJ software.

#### 3.2.8 Statistical analysis

All the results were statistically analyzed and plotted by GraphPadPrism 9.0.0. One-way ANOVA was used to compare multiple groups of samples, and then Tukey method was used to compare any two groups of data. *p* value (*p* < 0.05) showed that the difference was statistically significant. The IC_50_ values were calculated by GraphPadPrism 9.0.0 and the results of nonlinear regression of IC_50_ were plotted.

## 4 Results

### 4.1 Results of network pharmacology

#### 4.1.1 Potential targets of ginsenosides Rb1, CK, and gastric cancer

According to the HIT database, 22 targets of ginsenoside Rb1 and 2 targets of ginsenoside CK were obtained. 34, 32, and 140 gastric cancer-related targets were obtained from the disgenet, malacards, and OMIM databases, respectively. The above search results were combined and duplicates were deleted, and a total of 189 gastric cancer-related targets were obtained (Supplementary Table S1).

#### 4.1.2 Screening of key targets of ginsenoside Rb1 and CK in the treatment of gastric cancer

**FIGURE 1:**
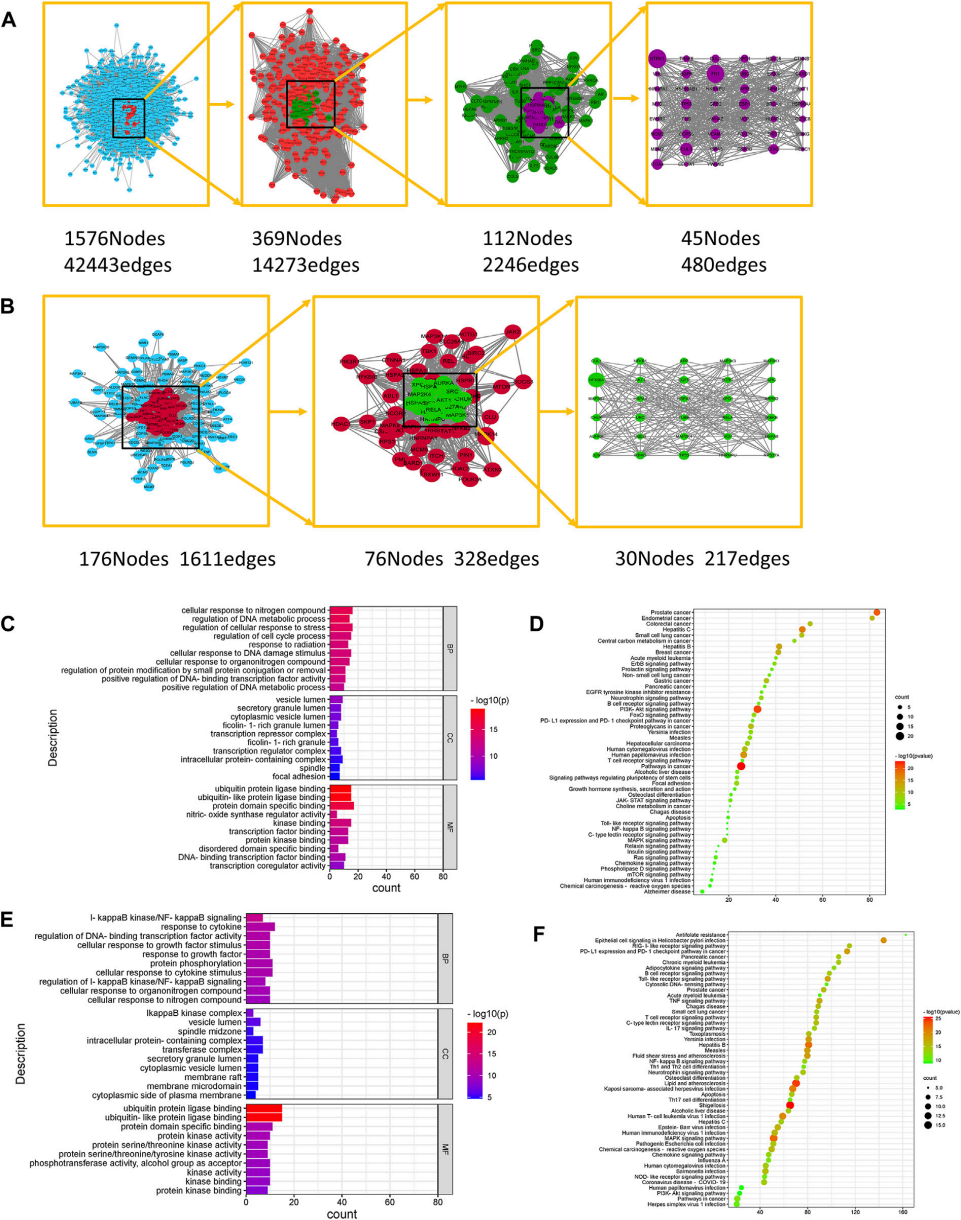
Network Pharmacological Analysis of Ginsenoside Rb1 and CK. **(A)** Topological network of key targets of ginsenoside Rb1 in the treatment of gastric cancer. **(B)** Topological network of key targets of ginsenoside CK in the treatment of gastric cancer. **(C)** GO biological function enrichment analysis of key targets of ginsenoside Rb1 in the treatment of gastric cancer. **(D)** KEGG pathway enrichment analysis of key targets of ginsenoside Rb1 in the treatment of gastric cancer. **(E)** GO biological function enrichment analysis of key targets of ginsenoside CK in the treatment of gastric cancer. **(F)** KEGG pathway enrichment analysis of key targets of ginsenoside CK in the treatment of gastric cancer.

The PPI network of ginsenoside Rb1 targets was constructed, including 2,083 nodes and 49,806 relationships between nodes; a PPI network of gastric cancer disease-related targets was constructed, including 5,804 nodes and 149,080 relationships between nodes. Then, using the Merge plug-in, the intersection targets of ginsenoside Rb1 in the treatment of gastric cancer were extracted, and then the key targets of ginsenoside Rb1 in the treatment of diseases were screened, such as Neurotrophic receptor tyrosine kinase 1 (NTRK1), Fibronectin 1 (FN1), Minichromosome maintenance complex component 2 (MCM2), Inhibitor of nuclear factor kappa B kinase subunit gamma (IKBKG), AKT serine/threonine kinase 1 (AKT1) and other 45 targets, as shown in Figure 1A.

The PPI network of the targets of ginsenoside CK was constructed, including 223 nodes and 1912 interrelationships between nodes; the PPI network of gastric cancer disease-related targets was constructed, including 5,804 nodes and 149080 interrelationships between nodes. Then the Merge plug-in to extract the intersection targets of ginsenoside CK in the treatment of gastric cancer was applied, and the key targets of ginsenoside CK in the treatment of diseases were screened, such as Heterogeneous nuclear ribonucleoprotein U (HNRNPU), Aurora kinase A (AURKA), IKBKB, AKT1 and so on, as shown in Figure 1B.

#### 4.1.3 Gene ontology and kyoto encyclopedia of genes and genomes analysis of key targets of ginsenoside Rb1 and CK

The key targets of ginsenoside Rb1 were analyzed by Gene Ontology (GO) using Metascape database, including 632 biological processes (BP), 53 cellular components (CC) and 72 molecular functions (MF). The enriched top 10 BP, CC, and MF are visualized as shown in Figure 1C. Among them, the biological process includes the regulation of cell cycle process, cell response to nitrogen compounds, regulation of DNA metabolism process, cell response to DNA damage stimulation, regulation of protein modification through small protein binding or removal, etc. Cell components involve cytoplasmic vesicle cavity, transcriptional inhibitory complex, intracellular protein complex, spindle and adhesion spot, etc. Molecular functions include protein domain specific binding, kinase binding, protein kinase binding, disordered domain specific binding, DNA binding transcription factor binding, transcription coregulator activity and so on. The results of Kyoto Encyclopedia of Genes and Genomes (KEGG) analysis of ginsenoside Rb1 key targets showed that the key targets of ginsenoside Rb1 were enriched to make a bubble map (Figure 1D), including Phosphatidylinositol 3-kinase/Protein kinases B (PI3K-AKT), FoxO signal pathway, ErbB s, JAK-STAT Ras signal pathway, Mammalian target of rapamycin (mTOR), Mitogen activated protein kinases (MAPK), Nuclear factor kappa-B (NF-κB), Toll-like receptor, and Wnt signal pathways.

The key targets of ginsenoside CK were analyzed by GO using Metascape database, which included 403 biological processes, 47 cellular components and 41 molecular functions. The enriched top 10 BP, CC, and MF are visualized as shown in Figure 1E. Among them, the biological process includes IκB kinase/NF-κB signal transduction, cell response to growth factor stimulation, protein phosphorylation, IκB kinase/NF-κB signal regulation, cell response to organic nitrogen compounds, etc. Cell components involve IκB kinase complex, vesicular cavity, spindle middle region, intracellular protein complex, membrane microdomain, etc. Molecular functions include ubiquitin protein ligase binding, protein kinase activity, protein serine/threonine kinase activity, phosphotransferase activity of alcohol group as receptor, kinase activity, kinase binding and so on. The KEGG analysis results of the key targets of ginsenoside CK are shown in Figure 1F, including PI3K-AKT signal pathway, MAPK signal pathway, NF-κB signal pathway, Toll-like receptor signal pathway, Wnt signal pathway, Rap1 signal pathway and so on.

As the purpose of this study was to compare the efficacy of ginsenoside Rb1 and CK in the treatment of gastric cancer, the common potential pathways of ginsenoside Rb1 and ginsenoside CK obtained from network pharmacology were studied. After the non-common pathway and non-cancer-related pathway were removed, the PI3K-AKT signal pathway, MAPK signal pathway, NF-κB signal pathway, Toll-like receptor signal pathway, TNF signal pathway, Wnt signal pathway, NOD-like receptor signal pathway, IL-17 signal pathway, HIF-1 signal pathway and so on were screened out. According to the GO analysis, the biological process involved in the anti-gastric cancer effects of ginsenoside Rb1 included the regulation of cell cycle process, the regulation of DNA metabolic process and the response of cells to DNA damage stimulation, which were related to cell apoptosis and proliferation. The biological process of ginsenoside CK was related to IκB kinase/NF-κB signal transduction, protein phosphorylation and the regulation of IκB kinase/NF-κB signal, which meant that the anti-gastric cancer effect of ginsenoside CK might be closely related to the IκB kinase/NF-κB signal pathway. Therefore, based on the biological process characteristics of ginsenoside Rb1 and CK obtained from GO analysis, it was found that PI3K/AKT/NF-κB might play an important role in the anti-gastric cancer effects of ginsenoside Rb1 and CK.

### 4.2 The results of the anti-cancer effects and mechanisms of ginsenoside Rb1 and CK

#### 4.2.1 Ginsenoside CK, rather than ginsenoside Rb1, inhibited the proliferation of gastric cancer cell line HGC-27

**FIGURE 2:**
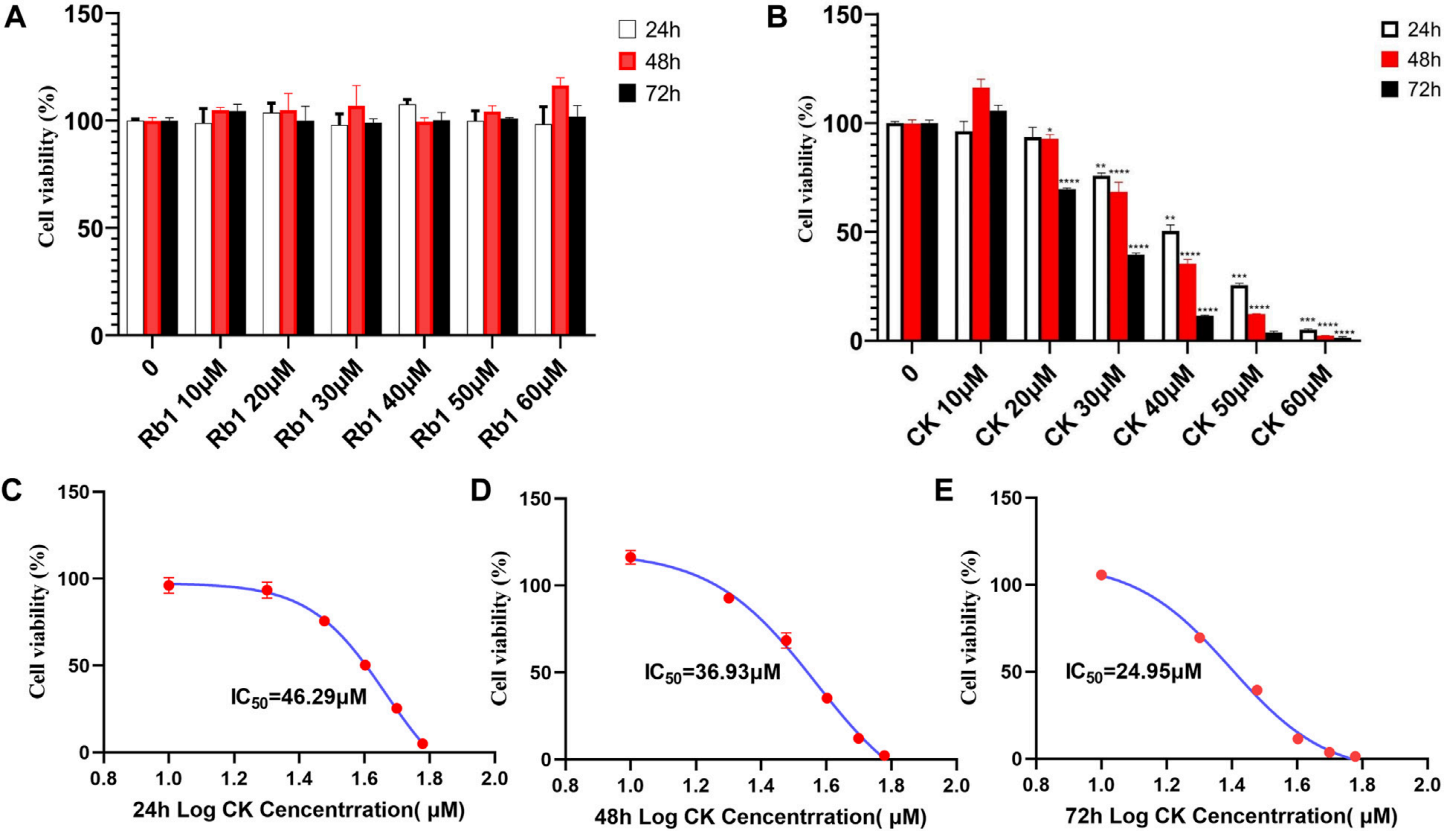
Effects of ginsenoside Rb1 and CK on Proliferation of Human Gastric cancer cells. **(A)** Effects of different concentrations of ginsenoside Rb1 and CK on the growth inhibition rate of human gastric cancer cells HGC-27 under different action times. **(B)** Effects of different concentrations of ginsenoside CK on the growth and survival rate of human gastric cancer cells HGC-27 under different action times. **(C)** Nonlinear regression results of IC_50_ after 24 h of CK treatment. **(D)** Nonlinear regression results of IC_50_ after 48 h of CK treatment. **(E)** Nonlinear regression results of IC_50_ after 72 h of CK treatment.

As was shown in Figures 2A,B, ginsenoside Rb1 had almost no inhibitory effect on the proliferation of HGC-27 cells. In contrast, ginsenoside CK had an inhibitory effect on the proliferation of HGC-27 cells. The inhibitory effect of ginsenoside CK on HGC-27 cells was concentration- and time-dependent. In addition, IC_50_ of ginsenoside CK in HGC-27 cells at 24, 48, and 72 h were 46.29, 36.93, and 24.95 μM as illustrated in the Figures 2C–E, respectively.

#### 4.2.2 Ginsenoside CK, rather than ginsenoside Rb1, reversed the morphological injury of HGC-27 cells

**FIGURE 3:**
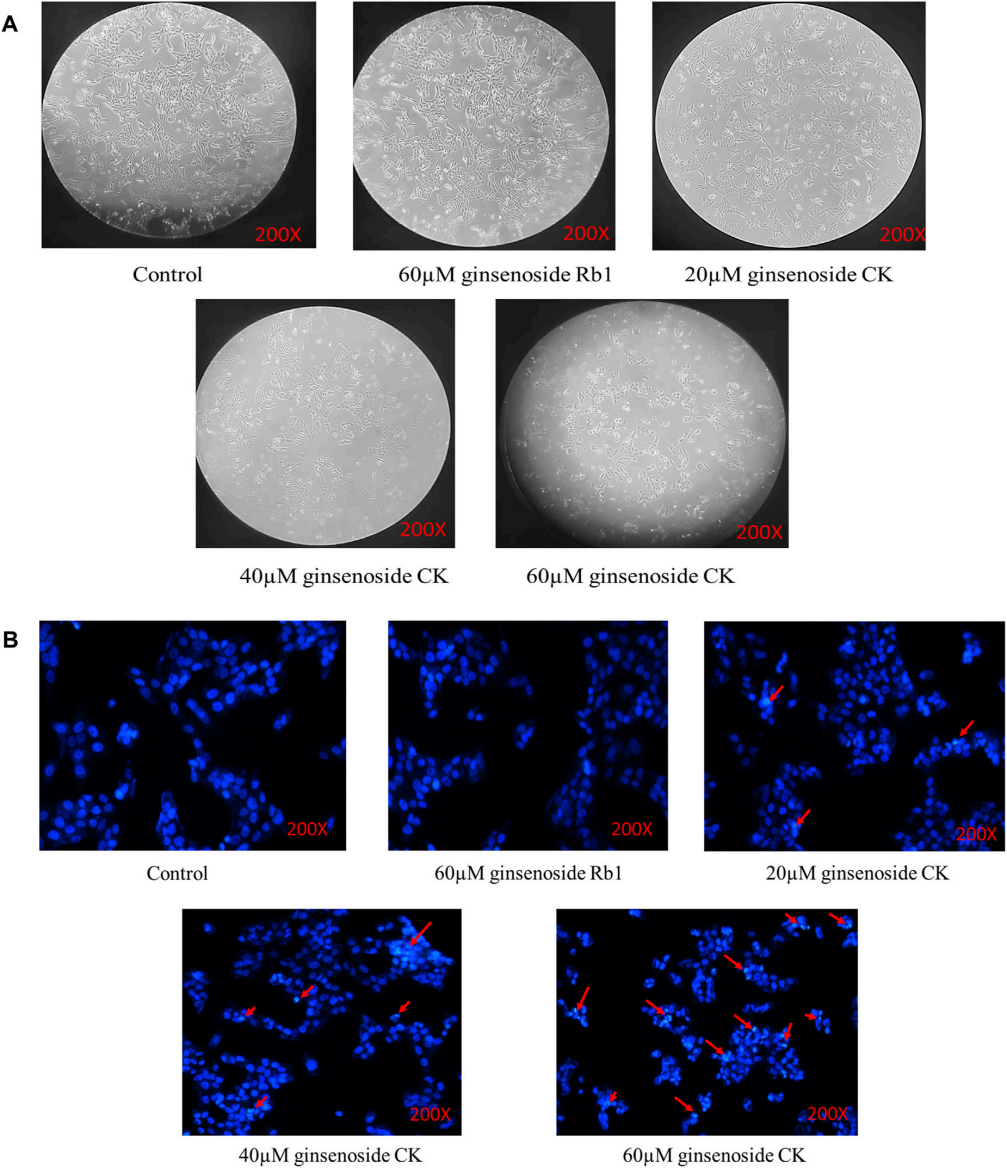
Effects of ginsenoside Rb1 and CK on the morphology and apoptosis of HGC-27 cells **(A)** Changes of cell morphology in each group after drug treatment. **(B)** Hoechst33258 staining results.

The normal adherent growth of cells in the blank group was observed under ×200 microscope, and the 60 μM ginsenoside Rb1 group and 20 μM ginsenoside CK group also grew well and distributed evenly without excessive inhibition as illustrated in the Figure 3A. However, the proliferation of cells in 40 μM ginsenoside CK and 60 μM ginsenoside CK was significantly inhibited, and the number of cells was lower than that in normal group. Not only that, the morphology of the two groups of cells also changed, showing shrinkage and clumps, and even obvious cell death. And with the increase of the dose of ginsenoside CK, the morphological abnormality was more obvious.

#### 4.2.3 Ginsenoside CK, rather than ginsenoside Rb1, induced apoptosis in HGC-27 cells

According to Figure 3B, Hoechst33258 staining results showed that the cells in the blank group and 60 μM ginsenoside Rb1 were light blue, and the distribution of chromatin is relatively uniform. After treated by 20, 40, and 60 μM ginsenoside CK, some cells were densely stained bright blue and showed nuclear fragmentation (red arrow), indicating the occurrence of apoptosis. And with the increase of the dose of ginsenoside CK, the number of normal cells decreased, and the characteristics of apoptosis were more obvious.

#### 4.2.4 Ginsenoside CK, rather than ginsenoside Rb1, inhibits the levels of cell cycle related proteins cyclinB1 and cyclinD1

**FIGURE 4:**
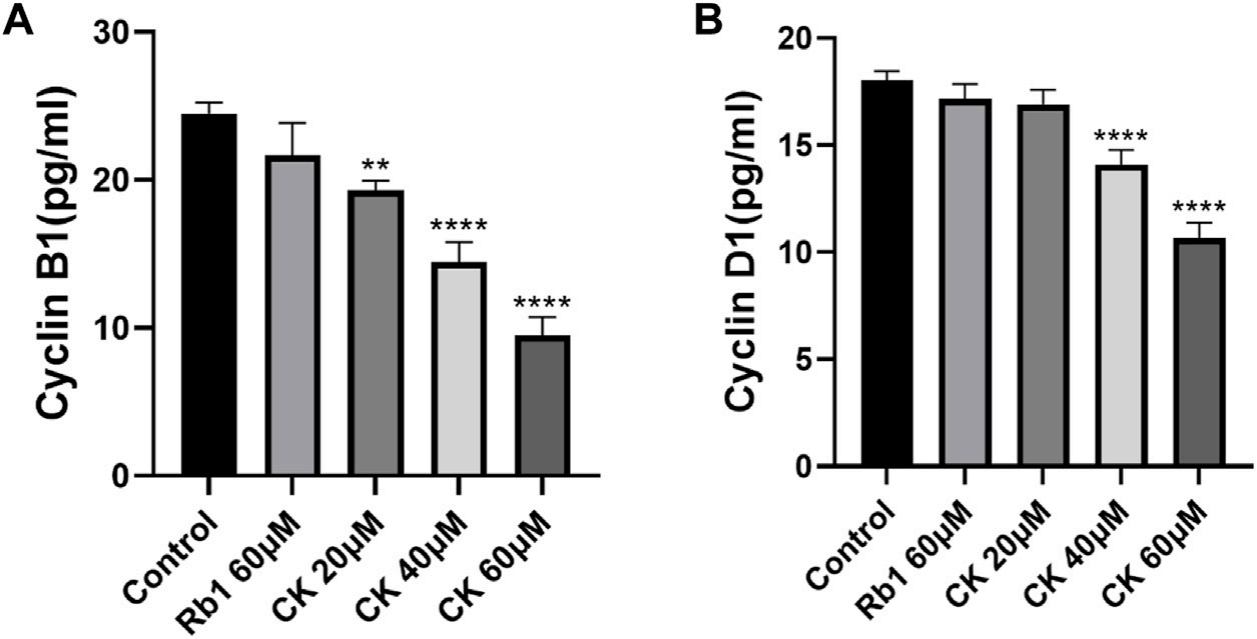
Effects of Ginsenoside Rb1 and Ginsenoside CK on Cyclin in HGC-27 **(A)** Histogram comparing the expression of CyclinB1 in each group. **(B)** Histogram comparing the expression of CyclinD1 in each group.

The results of Enzyme-linked immunosorbent assay (ELISA) (Figures 4A,B) showed that although ginsenoside Rb1 could downregulate cyclin CyclinB1, there was no significant difference between the control group and the control group. Ginsenoside CK could effectively downregulate the level of cyclin CyclinB1. Among them, 20, 40, and 60 μM CK significantly decreased the expression of CyclinB1 (*p* < 0.05).

In addition, compared with the blank group, ginsenoside Rb1 could only regulate cyclin CyclinD1 level in a down trend without significance (*p* > 0.05). Ginsenoside CK could effectively downregulate the level of cyclin CyclinD1 (*p* < 0.05). Among them, 40 and 60 μM CK significantly decreased the expression of CyclinD1 (*p* < 0.05).

#### 4.2.5 Ginsenoside CK, rather than ginsenoside Rb1, inhibited the expression of anti-apoptosis related protein Bcl-2 and promoted the expression of pro-apoptosis proteins Bax and Caspase 3 in HGC-27 cells

**FIGURE 5:**
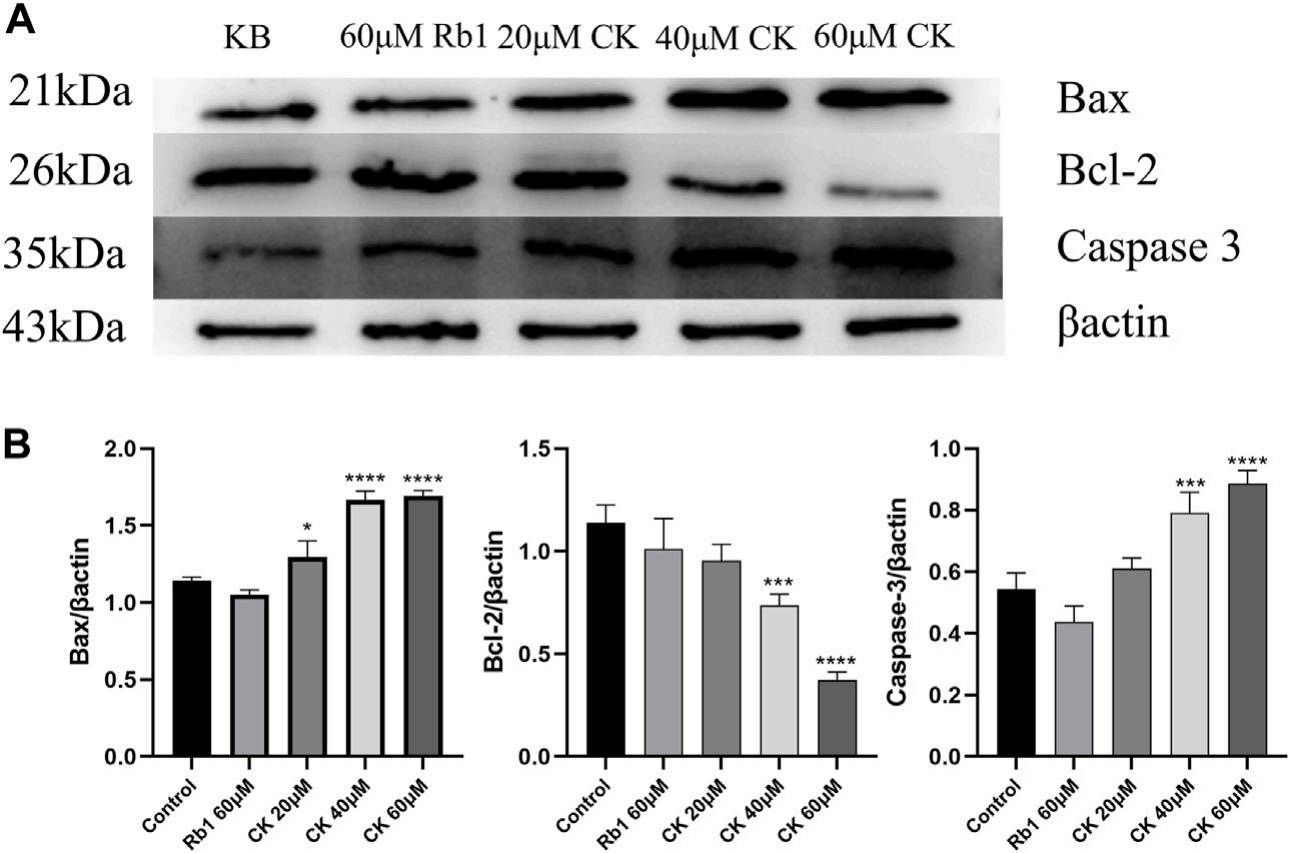
Effects of Ginsenoside Rb1 and Ginsenoside CK on Apoptosis-related Proteins in HGC-27 **(A)** Apoptosis-related proteins Bax, Bcl-2, Caspase 3 bands. **(B)** Histogram comparing the expressions of apoptosis-related proteins Bax, Bcl-2, and Caspase 3.

As can be seen from Figures 5A,B, ginsenoside Rb1 did not increase the expression of pro-apoptotic proteins Bcl-2 Associated X Protein (Bax) and Caspase 3 compared with the blank group. In addition, compared with the blank group, ginsenoside Rb1 could only downregulate the expression of anti-apoptotic protein B-cell lymphoma-2 (Bcl-2) in a down trend, without significant significance (*p* > 0.05). In contrast, ginsenoside CK could effectively increase the expressions of pro-apoptotic proteins (*p* < 0.05). Among them, 40 and 60 μM ginsenoside CK could significantly increase the expression of Caspase 3 (*p* < 0.05), and 20, 40, and 60 μM ginsenoside CK significantly increased the expression of Bax (*p* < 0.05). In addition, ginsenoside CK decreased the expression of Bcl-2, especially at the concentration of 40 and 60 μM (*p* < 0.05).

#### 4.2.6 Ginsenoside CK, rather than ginsenoside Rb1, inhibited the protein expression of PI3K/AKT/NF-κB in HGC-27 cells

**FIGURE 6:**
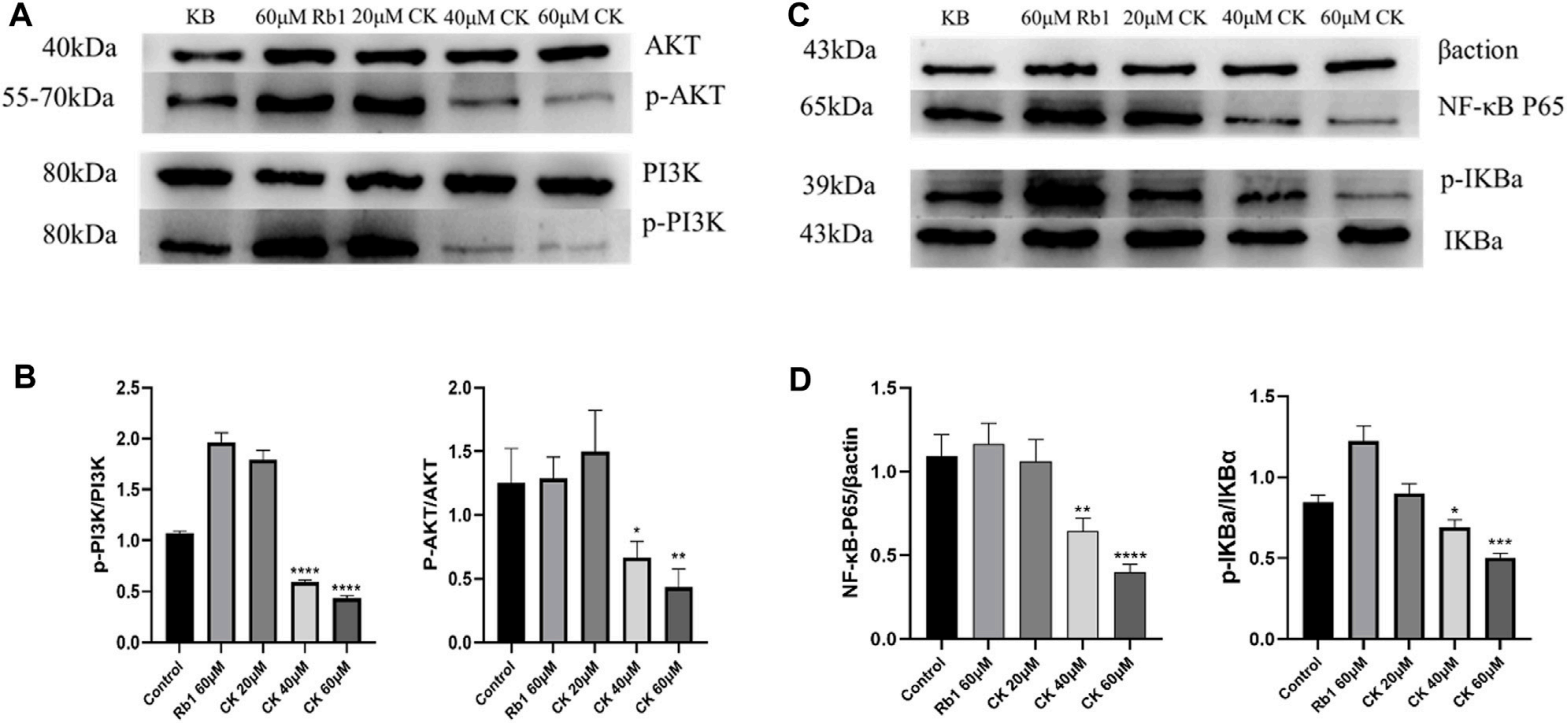
Effects of Ginsenoside Rb1 and Ginsenoside CK on Apoptosis-related Pathways Proteins in HGC-27 **(A)** Apoptosis-related pathways PI3K, p-PI3K, AKT, p-AKT bands. **(B)** Histogram comparing the expression of p-PI3K and p-AKT proteins in apoptosis-related pathways. **(C)** Apoptosis-related pathways NF-κB P65, Inhibitor *α* of NF-κb (IκBα), p-IκBα bands. **(D)** Histogram comparing the expression of NF-κB P65 and p-IκBα in apoptosis-related pathways.

As illustrated in Figures 6A,B, ginsenoside Rb1 did not reduce the expressions of p-PI3K and p-AKT proteins compared with the blank group. In contrast, 40 and 60 μM ginsenoside CK significantly decreased the expressions of p-PI3K and p-AKT protein, indicating that middle and high doses of ginsenoside CK inhibited the activation of PI3K/AKT pathway.

Additionally, as shown in Figures 6C,D, compared with the blank group, ginsenoside Rb1 could not reduce the protein expressions of NF-κB p65 and p-I κBα, but promoted the protein expressions of NF-κB p65 and p-IκB *α*. In contrast, 40 and 60 μM ginsenoside CK significantly decreased the expressions of NF- κB p65 and p-I κB *α* protein, which suggested that middle and high doses of ginsenoside CK inhibited the activation of NF-κB pathway.

#### 4.2.7 Ginsenoside CK, rather than ginsenoside Rb1, decreased the mRNA level of PI3K/AKT/NF-κB

**FIGURE 7:**
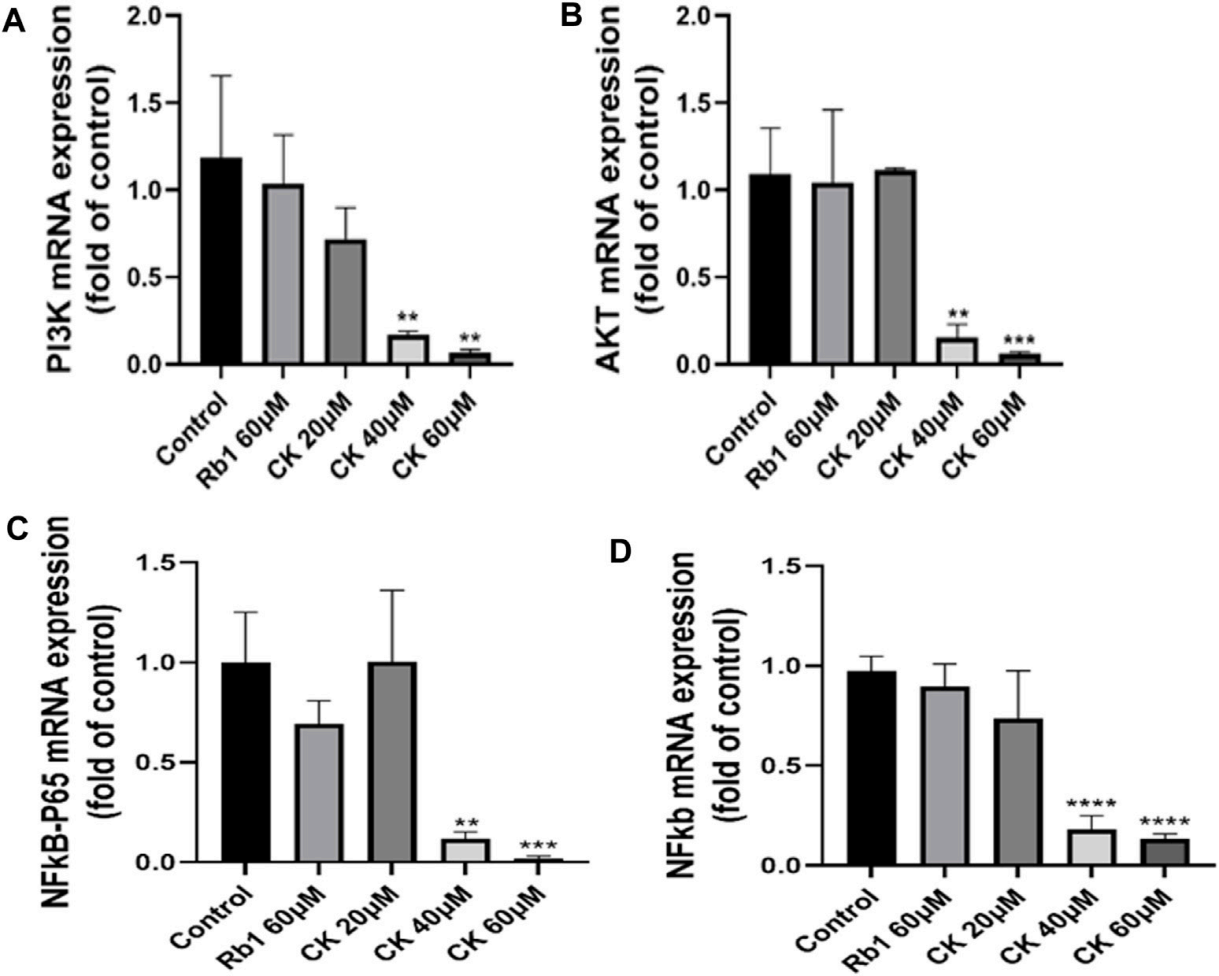
Effects of Ginsenoside Rb1 and Ginsenoside CK on apoptosis-related pathways mRNA in HGC-27 **(A)** Histograms comparing the expressions of PI3K mRNA in apoptosis-related pathways. **(B)** Histograms comparing the expressions of AKT mRNA in apoptosis-related pathways. **(C)** Histograms comparing the expressions of NF-κB p65 mRNA in apoptosis-related pathways. **(D)** Histograms comparing the expressions of NF-κB mRNA in apoptosis-related pathways.

Compared with the blank group, ginsenoside Rb1 did not significantly downregulate the mRNA expressions of PI3K and AKT as shown in Figures 7A,B. In contrast, ginsenoside CK at 40 and 60 μM could significantly reduce the mRNA expressions of PI3K and AKT, which further confirmed that middle and high doses of ginsenoside CK could also inhibit PI3K/AKT pathway at the gene levels.

As indicated in Figures 7C,D, compared with the blank group, ginsenoside Rb1 could only downregulate the mRNA expressions of NF-κB p65 and NF-κB in a down trend, without significant significance (*p* > 0.05). In contrast, 40, 60 μM ginsenoside K could significantly reduce the mRNA expressions of NF-κB p65 and NF-κB, which further confirmed that middle and high doses of ginsenoside CK could also inhibit NF-κB pathway at the transcriptional level.

## 5 Discussion

Like most herbal medicines, ginseng is generally consumed orally. When ginseng is administrated orally, its bioavailability is low due to incomplete parent compound absorption and conversion to metabolites. In the intestine, the main metabolic pathway consists of the deglycosylation of ginsenosides (including ginsenoside Rb1) in the intestinal microbiota by progressive cleavage of the sugar fraction (Tawab et al., 2003; Hasegawa, 2004; Liu et al., 2009). Previous studies showed that after ginseng ingestion, Rb1 is converted in the intestine to CK, which is the main metabolite absorbed into the body circulation (Tawab et al., 2003; Qi et al., 2011). As a parent compound, ginsenoside Rb1 itself does not have significant anticancer effects. In contract, ginsenoside CK showed significant anti-proliferative effects in gastric cancer cells (Hu et al., 2012). However, comparative studies on the anti-proliferative effects of ginsenoside CK and Rb1 in human gastric cancer cell lines have not been reported. Therefore, in this study, ginsenoside CK, instead of Rb1, had significant anti-gastric cancer proliferative activity, which was consistent with that of the cell morphological observations. Additionally, hoechst33258 staining results showed that ginsenoside CK induced cell apoptosis in gastric cancer cells treated by ginsenoside CK. ELISA results indicated that ginsenoside CK could effectively downregulate the levels of cyclinB1 and cyclinD1, and western blot results showed that CK inhibited the expression of anti-apoptosis-related protein Bcl-2 and promoted the expression of pro-apoptotic proteins Bax and Caspase 3. The above results tentatively confirmed that ginsenoside CK, the intestinal flora transformant of Rb1, had significant anti-gastric cancer activity but Rb1 showed no any effects, which was similar to the results of Rb1 and CK in colorectal cancer assays (Wang et al., 2012).Network pharmacology was a new discipline based on the theory of systems biology, which analyzed the network of biological systems and selected specific signal nodes for multi-target drug molecular design (Zhang et al., 2019; Wang et al., 2021). In this study, network pharmacology was used to predict the related targets of ginsenoside Rb1 and ginsenoside CK in the treatment of gastric cancer. The biological process of ginsenoside Rb1 obtained by GO analysis was close to cell apoptosis and proliferation. The biological process of ginsenoside CK was closely related to IκB kinase/NF-κB signal pathway. KEGG results showed that the common potential pathways of ginsenoside Rb1 and ginsenoside CK included PI3K-AKT signal pathway, MAPK signal pathway, NF-κB signal pathway, Toll-like receptor signal pathway, TNF signal pathway, Wnt signal pathway and so on. Inhibition of PI3K/AKT/NF-κB pathway was a potential target for cancer treatment (Sha et al., 2014; Pickard et al., 2017; Mao et al., 2019; Lian et al., 2020; Ling et al., 2020; Sun et al., 2020; Zhang et al., 2020). In the study of gastric cancer apoptosis induced by traditional Chinese medicine or compound prescription of traditional Chinese medicine, PI3K/AKT/NF-κB signal pathway axis of apoptosis-related pathway played an important role in the occurrence and development of gastric cancer (Sha et al., 2014). PI3K/AKT pathway is a classic pathway for activating NF-κB. It has been found in most tumors and used as a target for drug therapy. Studies on gastric cancer cells have shown that after regulation of AKT signal pathway, the expressions of Bax and Bcl-2 proteins can be regulated and apoptosis occurs (Wu et al., 2013; Gou et al., 2015; Wang et al., 2015; Niapour and Seyedasli, 2022). NF-κB plays an important role in regulating cellular response because it is a “fast acting” primary transcription factor that can be activated without new protein synthesis. When NF-κB is activated by corresponding stimulators, expressions of Bax, caspase-3 increased and Bcl-2 are decreased, showing a role in promoting apoptosis (Sun et al., 2014; Li et al., 2015; Rui et al., 2016; Xu et al., 2018b; Shang et al., 2019). Therefore, the authors believed that ginsenoside Rb1 and CK might play a role in the treatment of gastric cancer by inhibiting PI3K/AKT/NF-κB pathway and inducing apoptosis of gastric cancer cells. However, the current network pharmacology results were only conjectures and still needed to be validated by experiments, especially *in vitro* experiments. Therefore, according to the results of network pharmacology, the effect of ginsenoside Rb1 and CK on apoptosis-related pathway PI3K/AKT/NF-κB were investigated. In this study, the levels of NF-κB and PI3K/AKT related proteins were detected by Western blotting and RT-qPCR methods. The results showed that ginsenoside CK could inhibit the PI3K/AKT and NF-κB pathways in both of transcriptional and translational levels, whereas ginsenoside Rb1 had no obvious inhibitory effect.

To sum up, this experiment combines network pharmacology with *in vitro* cell experiment to study the drug action mechanism from the point of view of multi-target, optimizes the complex process of multi-target drug design. The results indicated that ginsenoside Rb1 had no anti-gastric cancer activity, while the intestinal microbiota metabolite of ginsenoside Rb1, ginsenoside CK could effectively exert its anti-gastric cancer activity *in vitro*. Moreover, ginsenoside CK, rather than ginsenoside Rb1, could induce cell apoptosis, and regulate expressions of apoptosis related proteins and cycle-related factors. The underlying mechanism may be attributed to its inhibition of the P13K/Akt/NF-κB signaling pathway. Therefore, ginsenoside CK may be the *in vivo* material basis for the anti-gastric cancer activity of ginsenosides. In conclusion, our study provides a scientific basis for the rational clinical application of ginsenosides, and sheds light on the studies of oral traditional Chinese medicines with low bioavailability and excellent therapeutic effect.

## Data Availability

The original contributions presented in the study are included in the article/Supplementary Material, further inquiries can be directed to the corresponding author.
