# Supplementary figures and images for: Ginsenoside CK, rather than Rb1, possesses potential chemopreventive activities in human gastric cancer via regulating PI3K/AKT/NF-κB signal pathway
Source: Front Pharmacol. 2022 Sep 29;13:977539. doi: 10.3389/fphar.2022.977539 (PMC9556731; doi:10.3389/fphar.2022.977539)

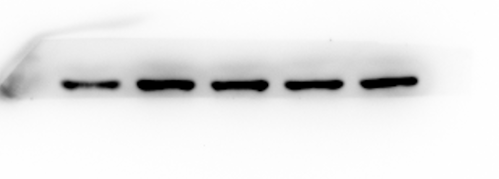

Supplement: Supplementary file 1 [file DataSheet1.ZIP › additional fiels/WB/AKT3.tif]

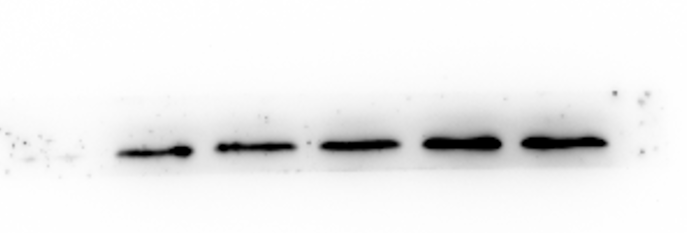

Supplement: Supplementary file 1 [file DataSheet1.ZIP › additional fiels/WB/bax.tif]

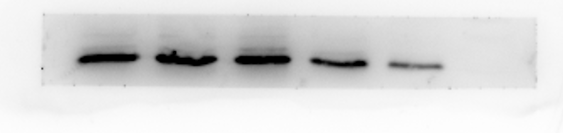

Supplement: Supplementary file 1 [file DataSheet1.ZIP › additional fiels/WB/Bcl-2.tif]

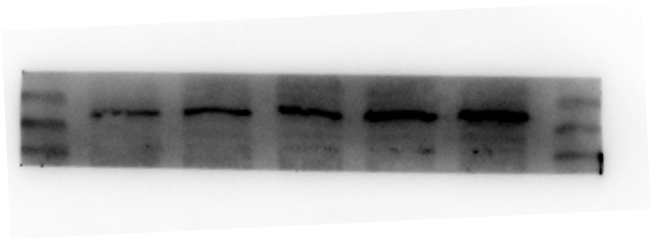

Supplement: Supplementary file 1 [file DataSheet1.ZIP › additional fiels/WB/caspase3.tif]

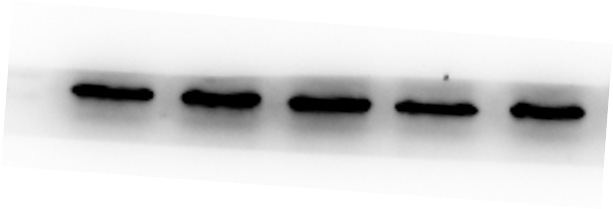

Supplement: Supplementary file 1 [file DataSheet1.ZIP › additional fiels/WB/IKBa.tif]

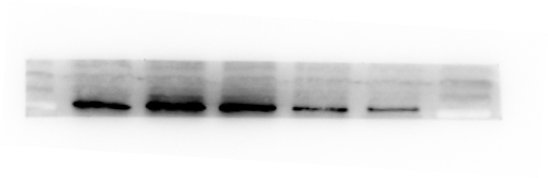

Supplement: Supplementary file 1 [file DataSheet1.ZIP › additional fiels/WB/NFkb-p65.tif]

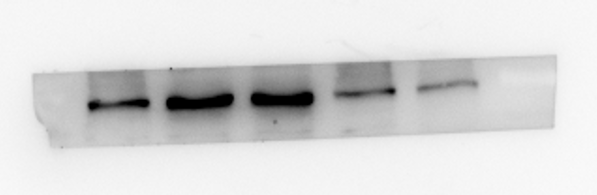

Supplement: Supplementary file 1 [file DataSheet1.ZIP › additional fiels/WB/P-AKT11.tif]

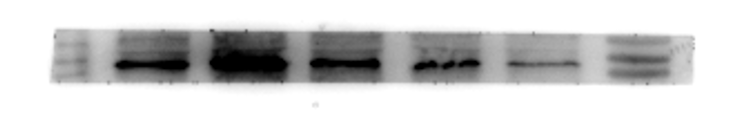

Supplement: Supplementary file 1 [file DataSheet1.ZIP › additional fiels/WB/p-ikba.tif]

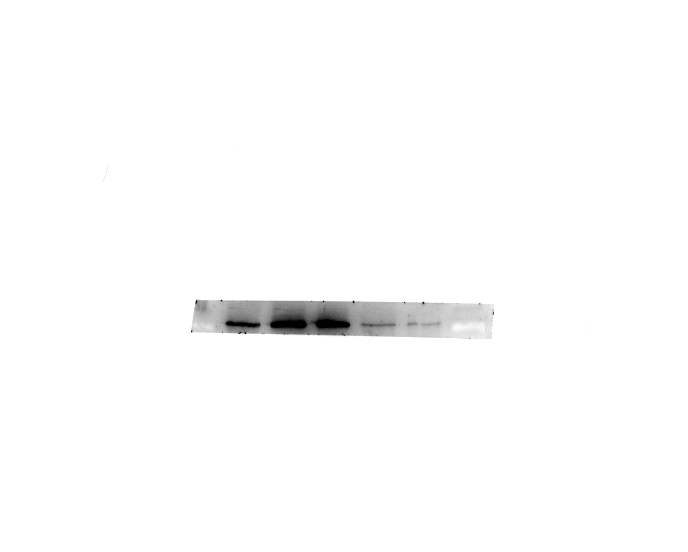

Supplement: Supplementary file 1 [file DataSheet1.ZIP › additional fiels/WB/p-pi3k.tif]

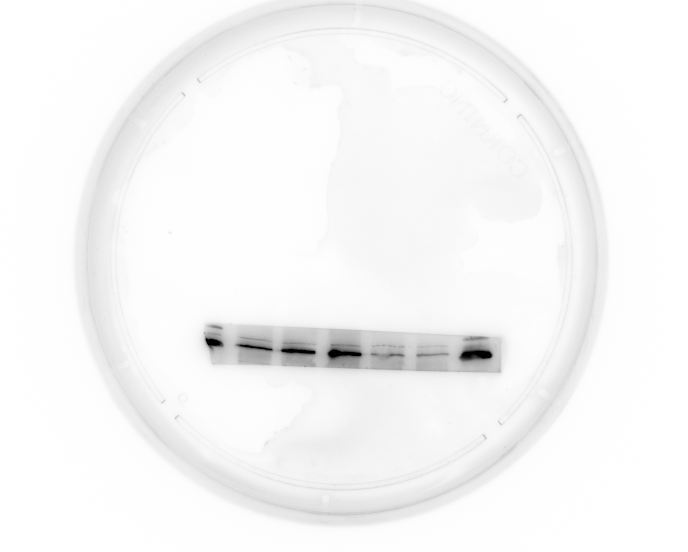

Supplement: Supplementary file 1 [file DataSheet1.ZIP › additional fiels/WB/p-pi3k2.tif]

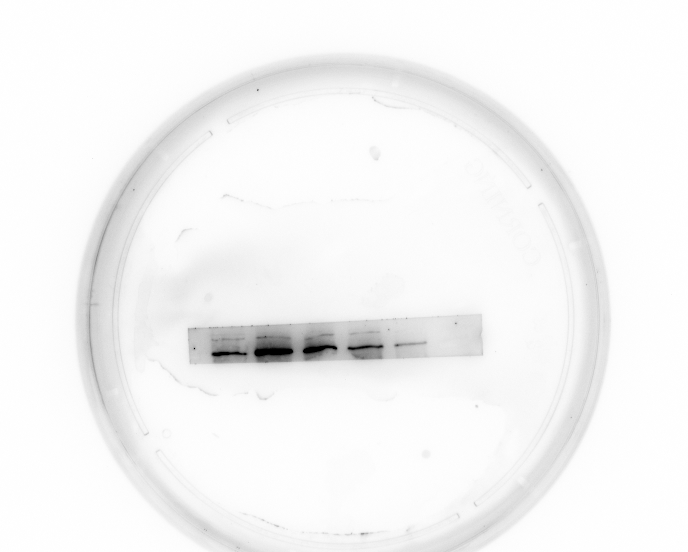

Supplement: Supplementary file 1 [file DataSheet1.ZIP › additional fiels/WB/p-pi3k4.tif]

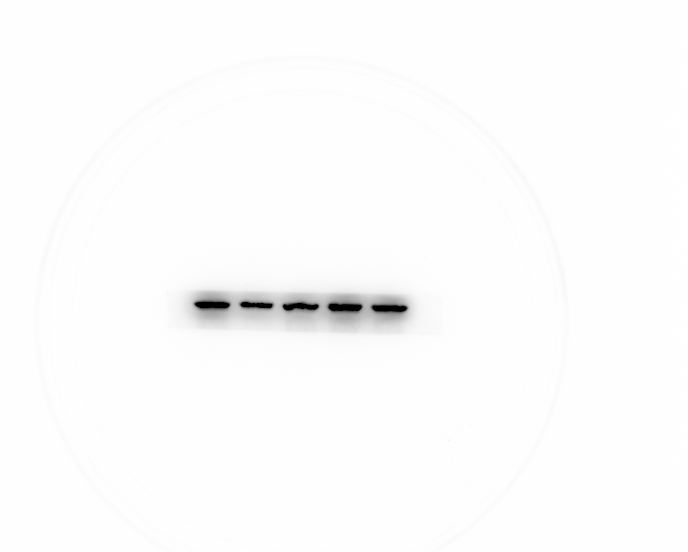

Supplement: Supplementary file 1 [file DataSheet1.ZIP › additional fiels/WB/pi3k9.tif]

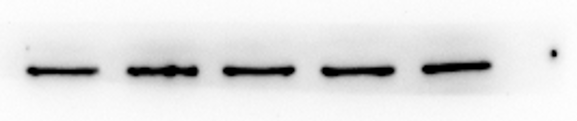

Supplement: Supplementary file 1 [file DataSheet1.ZIP › additional fiels/WB/βaction.tif]
