# Supplementary material for: Ginsenoside CK, rather than Rb1, possesses potential chemopreventive activities in human gastric cancer via regulating PI3K/AKT/NF-κB signal pathway
Source: Front Pharmacol. 2022 Sep 29;13:977539. doi: 10.3389/fphar.2022.977539 (PMC9556731; doi:10.3389/fphar.2022.977539)
Supplement: Supplementary file 3 [file DataSheet2.ZIP › Organized Western blot .docx]

### **1** **Ginsenoside CK, Rather Than Ginsenoside Rb1, inhibited the Expression of Anti-apoptosis related Protein Bcl-2 and Promoted the Expression of Pro-apoptosis Proteins Bax and Caspase3 in HGC-27 Cells**

**Bcl-2/βactin grayscale value**

**Control Rb1 60μM CK 20μM CK 40μM CK 60μM**

**1.057525 0.843239 0.860999 0.67799 0.331545**

**1.23087 1.126866 1.010678 0.752396 0.37275**

**1.125594 1.06266 0.987205 0.780736 0.411431**

**Bax/βactin grayscale value**

| **Control** | **Rb1 60μM** | **CK 20μM** | **CK 40μM** | **CK 60μM** |
| --- | --- | --- | --- | --- |
| **1.111487** | **1.081464** | **1.389715** | **1.702556** | **1.673665** |
| **1.156835** | **1.017175** | **1.183254** | **1.600217** | **1.668626** |
| **1.154117** | **1.053864** | **1.313833** | **1.697175** | **1.733604** |

**Caspase-3/βactin grayscale value**

| **Control** | **Rb1 60μM** | **CK 20μM** | **CK 40μM** | **CK 60μM** |
| --- | --- | --- | --- | --- |
| **0.502446** | **0.388101** | **0.575737** | **0.743067** | **0.861609** |
| **0.52654** | **0.43457** | **0.613334** | **0.767532** | **0.863192** |
| **0.602931** | **0.48981** | **0.643395** | **0.86639** | **0.935243** |

**NF-κB p65/βactin grayscale value**

| **Control** | **Rb1 60μM** | **CK 20μM** | **CK 40μM** | **CK 60μM** |
| --- | --- | --- | --- | --- |
| **0.944943** | **1.048233** | **0.933717** | **0.575557** | **0.361297** |
| **1.137192** | **1.296343** | **1.194302** | **0.730341** | **0.448738** |
| **1.190899** | **1.150695** | **1.054254** | **0.623792** | **0.393496** |

**p-PI3K /** **PI3K grayscale value**

| **Control** | **Rb1 60μM** | **CK 20μM** | **CK 40μM** | **CK 60μM** |
| --- | --- | --- | --- | --- |
| **1.084713** | **2.066351** | **1.692486** | **0.585602** | **0.461111** |
| **1.054588** | **1.875992** | **1.860738** | **0.612972** | **0.431561** |
| **1.085189** | **1.934721** | **1.832315** | **0.588314** | **0.417599** |

**p-AKT /** **AKT grayscale value**

| **Control** | **Rb1 60μM** | **CK 20μM** | **CK 40μM** | **CK 60μM** |
| --- | --- | --- | --- | --- |
| **1.003223** | **1.100409** | **1.2051** | **0.573426** | **0.410284** |
| **1.204306** | **1.333454** | **1.455089** | **0.62055** | **0.304619** |
| **1.543292** | **1.427895** | **1.840541** | **0.808609** | **0.587279** |

**p-** **IκBα / IκBα grayscale value**

| **Control** | **Rb1 60μM** | **CK 20μM** | **CK 40μM** | **CK 60μM** |
| --- | --- | --- | --- | --- |
| **0.79646** | **1.278368** | **0.941751** | **0.706332** | **0.471589** |
| **0.85735** | **1.115364** | **0.826065** | **0.635658** | **0.519033** |
| **0.880597** | **1.277743** | **0.925739** | **0.725661** | **0.516631** |

**Table 1 Expression of apoptosis-related proteins Bax, Bcl-2 and Caspase 3 ( ‾x±S）**

| **Group** | **Bax** | **Bcl-2** | **Caspase-3** |
| --- | --- | --- | --- |
| **Control** | **1.141±0.025** | **1.138±0.087** | **0.544±0.052** |
| **Rb1 60μM** | **1.051±0.032** | **1.011±0.149** | **0.436±0.051** |
| **CK 20μM** | **1.296±0.104^*^** | **0.953±0.081** | **0.611±0.034** |
| **CK 40μM** | **1.667±0.058^****^** | **0.737±0.053^***^** | **0.792±0.065^***^** |
| **CK 60μM** | **1.692±0.036^****^** | **0.372±0.040^****^** | **0.887±0.042^****^** |

**Tip：GP: 0.1234(ns), 0.0332(*), 0.0021(**),0.0002(***), 0.0001(****)vs. Control**


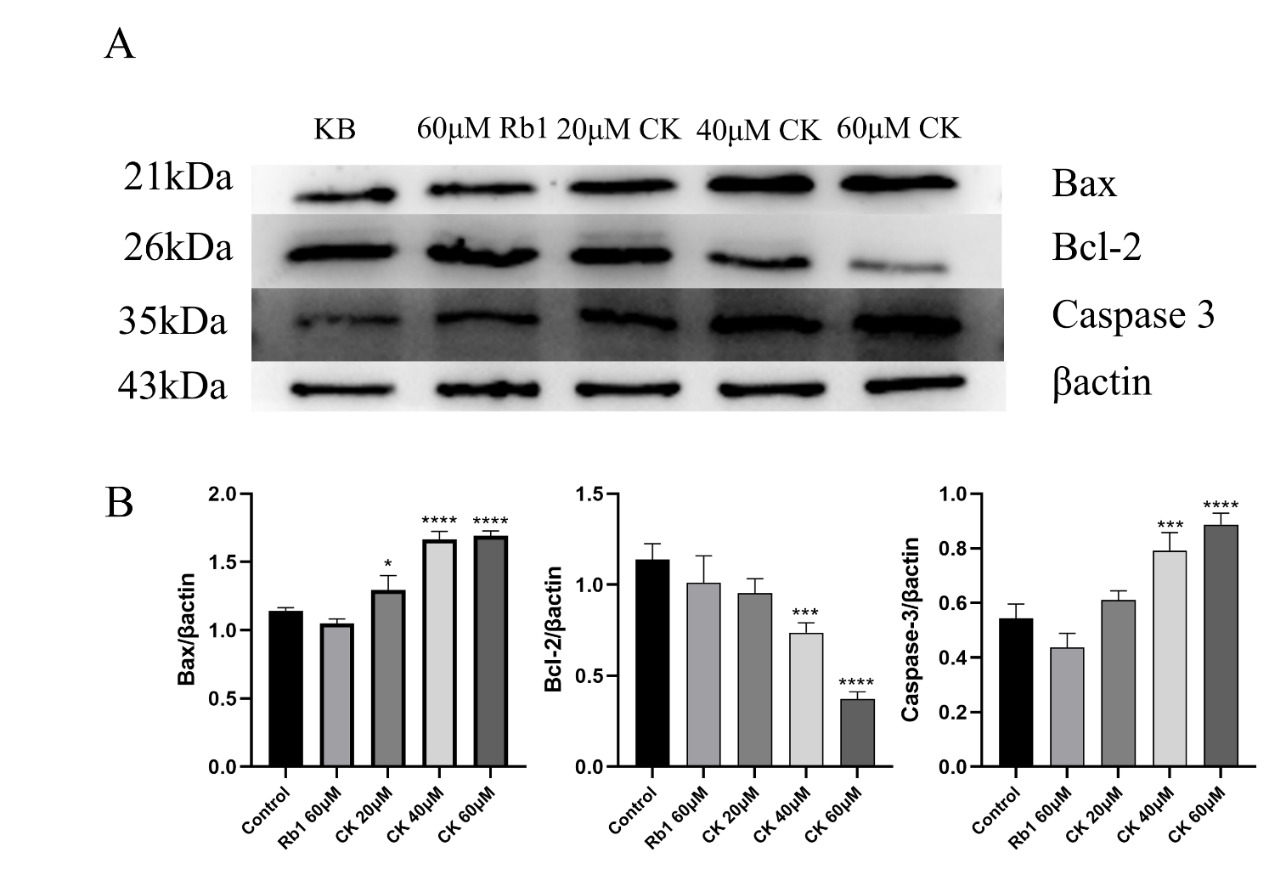


**Figure 5││ Effects of Ginsenoside Rb1 and Ginsenoside CK on apoptosis-related proteins in HGC-27** (A) Apoptosis-related proteins Bax, Bcl-2, Caspase 3 bands. (B) Histogram comparing the expressions of apoptosis-related proteins Bax, Bcl-2, and Caspase 3.

### **2. Ginsenoside CK, Rather Than Ginsenoside Rb1, Inhibited the Protein Expression of PI3K/AKT/****NF-κB in HGC-27 Cells**

**Table 2 Expression of** **p-PI3K and p-AKT proteins in apoptosis-related pathways ( ‾x±S）**

| **Group** | **p-PI3K** | **p-AKT** |  |
| --- | --- | --- | --- |
| **Control** | **1.075±0.018** | **1.250±0.273** |  |
| **Rb1 60μM** | **1.959±0.097** | **1.287±0.169** |  |
| **CK 20μM** | **1.795±0.104** | **1.500±0.32** |  |
| **CK 40μM** | **0.596±0.015^****^** | **0.668±0.124^*^** |  |
| **CK 60μM** | **0.437±0.022^****^** | **0.434±0.143^**^** |  |

**Table 3 Expressions of NF-κB P65 and p-IκBα proteins in apoptosis-related pathways ( ‾x±S）**

| **Group** | **NF-κB P65** | **p-** **IκBα** |  |
| --- | --- | --- | --- |
| **Control** | **1.091±0.125** | **0.845±0.043** |  |
| **Rb1 60μM** | **1.165±0.125** | **1.224±0.094** |  |
| **CK 20μM** | **1.061±0.130** | **0.898±0.063** |  |
| **CK 40μM** | **0.643±0.079^**^** | **0.689±0.047^*^** |  |
| **CK 60μM** | **0.401±0.044^****^** | **0.502±0.027^***^** |  |

**Tip：GP: 0.1234(ns), 0.0332(*), 0.0021(**),0.0002(***), 0.0001(****)vs. Control**


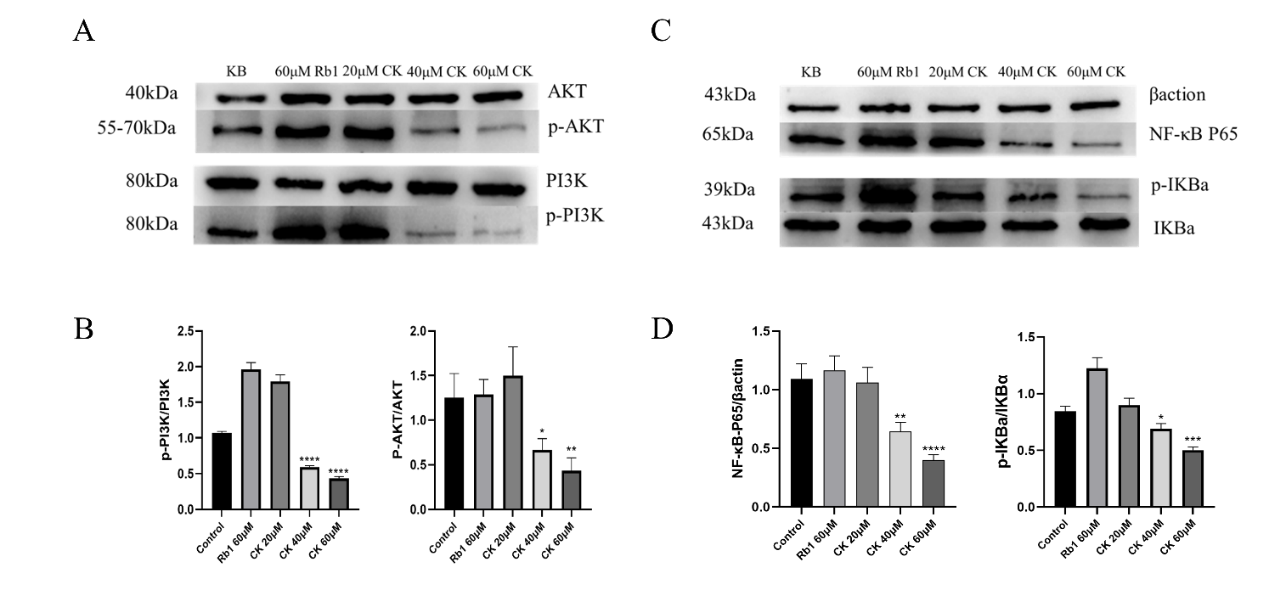


**Figure 6│Effects of Ginsenoside Rb1 and Ginsenoside CK on apoptosis-related pathways proteins in HGC-27** (A) Apoptosis-related pathways PI3K, p-PI3K, AKT, p-AKT bands. (B) Histogram comparing the expression of p-PI3K and p-AKT proteins in apoptosis-related pathways. (C) Apoptosis-related pathways NF-κB P65, Inhibitor α of NF-κb (IκBα), p-IκBα bands. (D) Histogram comparing the expression of NF-κB P65 and p-IκBα in apoptosis-related pathways.
